# The Gratitude of Hospital Patients

**Published:** 1887-01-01

**Authors:** 


### The Gratitude of Hospital Patients.

By a Practical Philanthropist.

In a reference to St. Thomas's Hospital the other day, I spoke incidentally of a handsome little mahogany bookcase, and 150 volumes of books that had been given by one who had been an inmate of that institution and desired to show his gratitude for the benefit he had received there.

Worthy of Imitation.

The allusion suggested an inquiry as to the frequency of such pleasant displays of right feeling, and as to the desirability of prompting patients to make them. A good many people find their way into the public hospitals not altogether because they are poor. They go there partly, perhaps, because it is convenient, and partly, no doubt, because they believe that, with all the resources of a great hospital for their benefit, they are likely to do better than in their own homes. One would be inclined to expect that a good many patients of this class would find a pleasure in making some little recognition of what has been done for them when they leave. I am not, of course, referring to "paying patients," though some of them not only pay the fair charges of the hospital, but go considerably beyond that, or supplement their payment by very generous gifts. Some little time ago, for instance, a gentleman was knocked down in the street and rather seriously injured. He was carried into the Middlesex Hospital, where he lay unable to be moved for some time, and afterwards preferred to remain until able to get about again. On leaving, he presented a cheque for £100. A patient at Guy's, after paying his bill, presented a dozen handsome little candle lamps, to be fixed one over each bed in the ward he had lain in, for the comfort of future patients who might desire to read in bed; and another gave £50 to be employed as the authorities might think best for the adornment or the comfort of the same department of the hospital. This generosity has permitted, among other things, of the provision of an electric bell-touch at each bed-head, a convenient bell-register in the nurses' hall, as well as the addition of a number of handsome gas-brackets.

But these are of course the gifts of comparatively wealthy people, and represent only a very small section of hospital inmates. The inquiry suggested had reference to the gratitude of those who availed themselves of hospital benefits and were not expected to pay. How often does it happen that they show their sense of obligation by some little gift? Truth to tell it is not very easy to say. At all the hospitals the wards have money-boxes for the contributions of those who may like to express their gratitude by dropping in such small sums as they may be able to afford, and how many contributions there are, and what proportion their offerings may bear to their means, it is impossible to say. I am afraid the most charitable calculation cannot make the proportion a very large one. There are 6,000 or 7,000 patients who pass through the beds at St. Bartholomew's in the course of a year, and last year the boxes there yielded only £28 6*s*. 2*d*. Take the number of in-patients during the year at 6,500, and this will give a little over a penny apiece; but if we add to the 6,500 in-patients the vastly greater number of out-patients, the average will be reduced to a microscopical fraction of a farthing. They had during the year, it is true, a few larger sums not put into the boxes, but sent by patients or the friends of patients as contributions to the general funds. One very satisfactory item of the kind was a cheque for £25 from the employés of Messrs. Humphreys and Tenant of Deptford, in acknowledgment, as I understand, of services rendered to some of their number. The husband of a patient sent fifteen guineas; two sisters of a patient sent five guineas; a patient himself presented five guineas, and there were one or two smaller sums. Altogether, there were six such contributions made in evidence of gratitude last year, and there were just the same number the year before. At Guy's, they had, last year, small contributions put into the boxes for the benefit of patients leaving hospital in a destitute state, about £35; and though Dr. Steel mentions the fact as if he considers it very creditable to the patients, I am afraid his opinion must be attributed mainly to the geniality and kindness of nature for which the Superintendent of Guy's is so distinguished, and which inclines him to look pleasantly on the lump sum rather than to split it up into so much per head. They have, however, at Guy's a few very nice presents that have been sent by patients as expressions of gratitude for kindness shown and health restored. Over one of the wooden doors is a large framed engraving of a well-known picture; in another are two bronze figures ornamenting the mantel-piece, and each conspicuously inscribed, "From a Grateful Patient." The same pleasant legend beams down from the dial of a large clock, and in different part of the hospital, they have expensive chairs which patients have found necessary for their own use after leaving the hospital, and which, when they have done with, they have presented to the institution where they have received so much to be thankful for. Minor gifts, such as scrap-albums, are also not infrequent here, and all the hospitals are continually receiving letters in which patients express themselves deeply sensible of the benefits they have received in their time of sickness and suffering. As for the children, the secretary of the Middlesex tells me that they often show their appreciation of hospital kindness and comfort by crying piteously when the time comes for

Where are the Nine?

them to be turned out. Here, by the way, they have a presented bookcase, and one quarter recently their money-boxes yielded as much as £20, and last year they had altogether £43 14*s*., made up of small sums given by patients or their friends.

No Begging from Patients.

Of course it must not be forgotten that the great majority of hospital patients are poor. They are, in fact, in hospital because they are poor, and, in a great many cases, while they are lying there sick and helpless, things are going all wrong at home. Income has stopped while expenses are running on, and it would be a great pity to do anything by way of adding to the trouble of the anxious and heart-burdened sick in hospital. To do anything which would be calculated to make uneasy any patient unable to present money or books or pictures on leaving, would be a great mistake ; and if it became an understood thing that all patients who could make presents were expected to do so, that would certainly be the effect on a good many inmates, and among them would be a large proportion of the more sensitive and conscientious. No doubt it is wise, therefore, to refrain, as all the hospitals appear to do, from any form of solicitation of gifts or contributions—except the provision of money-boxes, and they are for the most part for the benefit of needy patients when they leave. Any system of suggesting or soliciting gifts would be pretty sure to create in the minds of patients unable to afford them, a suspicion that they were treated with less consideration than those who could. It is, therefore, on several grounds undesirable that hospital authorities should do anything by way of evoking these little displays of gratitude, which, after all, are not in themselves of any very overwhelming importance. It is the moral rather than the material value of them which is of importance, and their moral worth is of course just in proportion as they are spontaneous and sincere expressions of good feeling. Viewed in this light they are very important ; and though it may be undesirable that hospital managers should take any means of eliciting practical displays of grateful feeling, it is nevertheless true that every little acknowledgment is not only creditable to the person who makes it, but tends to benefit other patients, and to engender kindly feeling among all concerned in these great and invaluable institutions.

---

Hair Parting.—A medical student has sent us a communication on what he deems the "very important" subject of hair parting. He parts his hair in the centre. This is the natural and proper fashion, he insists, and was adopted by Bolingbroke, Prince Rupert, Milton, and John Hampden, and he adds that Mr. Darwin tells us that the long-haired monkeys invariably part their hair in the middle also.

